# Comparison of depressive symptoms between heterosexual and sexual minority university students: network analysis

**DOI:** 10.1186/s41155-026-00388-z

**Published:** 2026-04-06

**Authors:** Elton Brás Camargo Júnior, Nelson Silva Rodrigues Júnior, Gilson Gonçalves Silva, Maria Neyrian de Fátima Fernandes, Edna Idalia Paulina Navarro-Oliva, Edilaine Cristina da Silva Gherardi-Donato

**Affiliations:** 1 Universidade de Rio Verde, Rio Verde, Brazil; 2 Federal University of Maranhão, Imperatriz, Brazil; 3 Facultad de Enfermería Dr. Santiago Valdés Galindo, Autonomous University of Coahuila, Saltillo, Mexico; 4 Escola de Enfermagem de Ribeirão Preto, Universidade de São Paulo, Ribeirão Preto, Brazil

**Keywords:** Depression, Network analysis, Heterosexuality, Sexual and gender minorities

## Abstract

**Background:**

Network analysis has emerged as a powerful approach for examining the complex interrelationships among psychiatric symptoms. In the context of depression, this method allows for identifying the most central symptoms that sustain the overall clinical picture, offering new possibilities for targeted interventions. Depression is a prevalent mental disorder that significantly affects university students, especially those belonging to sexual minorities. Understanding the interplay between depressive symptoms can inform more effective interventions by targeting the symptoms most central to the disorder.

**Objective:**

This study aimed to compare the interrelationship between depressive symptoms between groups of heterosexual and sexual minority students through network analysis.

**Methods:**

In this cross-sectional study, depressive symptoms were evaluated through the Patient Health Questionnaire-9 (PHQ-9) among 1,271 university students. A network analysis was then conducted to identify the most influential symptoms and their associated patterns, based on centrality indices.

**Results:**

The analysis revealed that sexual minority students reported significantly higher levels of depressive symptoms compared to their heterosexual students. In the network of heterosexual students, suicidal ideation showed the highest centrality, whereas feelings of guilt emerged as the most central symptom among sexual minority students.

**Conclusion:**

The findings suggest group-specific patterns of symptom centrality, with different symptoms appearing more central within each group. These patterns inform assessment and monitoring priorities in university settings.

**Supplementary Information:**

The online version contains supplementary material available at 10.1186/s41155-026-00388-z.

## Introduction

Depression ranks among the most prevalent psychiatric conditions globally, with an estimated 280 million individuals affected (Institute of Health Metrics and Evaluation, 2021). It is a multifactorial disorder with unclear triggering factors, representing a significant public health problem (Filatova et al., 2021). Understanding the psychopathological mechanisms involved in depression can reduce the risk of developing the disorder and contribute to the provision of effective treatment. Depressive disorder can emerge through dynamic interactions between symptoms, and this systemic perspective highlights depression as a constantly evolving interaction of symptomatic changes. Thus, depressive symptoms such as low mood, loss of interest in daily activities, and sleep problems, for example, can form a system of interrelated experiences (Malgaroli et al., 2021). This framework facilitates the use of network analysis to examine the reciprocal influences among symptoms, as each can potentially trigger or reinforce others (Cheung et al., 2021).

The network theory of psychopathology conceptualizes mental disorders as interactive systems of symptoms that directly influence each other, representing each symptom as a node within a network (Borsboom, 2017). Symptoms occupying more central positions in the network are considered influential, as their activation can increase the likelihood of other symptoms’ activation (Borsboom & Cramer, 2013). Network analysis, a recent theory and an advanced statistical approach, allows for the investigation of the interrelationships between symptoms. Through this lens, depression can be visualized as a web of interconnected symptoms, in which each symptom influences and is influenced by others. This technique provides a comprehensive perspective on mental disorders, going beyond traditional latent disease models to examine how symptoms dynamically contribute to the emergence and maintenance of the disorder (Fried, 2015). Findings from symptom networks have significant implications for understanding the development, persistence, and treatment of mental disorders (Gauld et al., 2023).

The application of network analysis to investigate depressive symptoms proves highly relevant in university populations, given that students are commonly exposed to numerous stressors, such as academic pressure, financial strain, social integration challenges, and career uncertainty, that may contribute to the onset and persistence of mental health disorders (Sheldon et al., 2021). The analytical approach, through the use of network analysis, also presents itself as an important tool for understanding the interrelationship of depressive symptoms when considering sexual minorities. In addition to the everyday challenges of the university environment, students from sexual minorities - a term that refers to individuals whose sexual orientation, gender identity, or gender expression diverges from the heterosexual and cisgender norm - are at greater risk of facing situations such as discrimination and stigma, which can intensify depressive symptoms and alter the way these symptoms connect (Balloo et al., 2022; Helminen et al., 2023).

To conceptualize these disparities, Minority Stress Theory describes how individuals belonging to sexual minorities are exposed to chronic and socially structured stressors such as stigma, discrimination, and internalized homophobia, which increase vulnerability to mental health problems (Frost & Meyer, 2023) and can significantly impact specific symptoms (e.g., guilt/shame) (Pachankis et al., 2024). In addition, the framework of psychological mediation suggests that these stressors operate through cognitive and affective mechanisms, including increased sensitivity to rejection, rumination, and negative self-evaluations (Hatzenbuehler, 2009). From a network analysis perspective, these stressors can function as external perturbations that activate or intensify the connectivity of specific depressive symptoms, potentially increasing their centrality and strengthening connections between cognitive and affective symptoms. Importantly, it is not sexual minority status per se that increases vulnerability, but rather the interaction between minority-related stressors and contextual factors, including academic demands and social environments, that may shape mental health outcomes.

Scientific evidence indicates that individuals from sexual minorities have higher rates of depression when compared to heterosexuals (Argyriou et al., 2021; Wittgens et al., 2022). In Brazil, although research analyzing the relationship between mental health and sexual or gender orientation is limited, the available studies demonstrate significant inequalities in the mental health of sexual minorities (Gomes & Lopes, 2023; Terra et al., 2022). At the same time, in recent years, there has been an increase in the number of studies that have used network analysis as a methodological approach to identify connections between depressive symptoms (Malgaroli et al., 2021). However, published studies have analyzed depressive symptom networks in the aggregate without exploring possible differences in symptom networks between people belonging to sexual minorities and heterosexuals (Brooks et al., 2025; Z. Chen et al. 2025a, 2025b; Liu et al. 2025; Zhao et al. 2023). As far as we are aware, studies employing network analysis to explore depressive symptoms in sexual minority populations remain limited, with existing investigations primarily focusing on women (Li et al., 2025).

### Current study

The gap in scientific evidence regarding the lack of network analysis studies of depressive symptoms in sexual minorities highlights the importance of exploring how depression manifests in groups with different sexual orientations. In this sense, the present study, by identifying the core depressive symptoms among groups of sexual minority and heterosexual university students, will provide results that can guide personalized interventionist practices to address mental health inequalities in these population groups. Thus, the study aimed to identify and compare the relationship between depressive symptoms of sexual minorities and heterosexual university students through network analysis. Based on minority stress and network theory frameworks, we hypothesized that sexual minority students would exhibit greater centrality in self-referential and affective symptoms (e.g., guilt, worthlessness, hopelessness), reflecting the impact of minority-related stress processes.

## Method

### Study design and subjects

This is a cross-sectional and exploratory study. This research was conducted with university students from an educational institution located in five cities in the state of Goiás, Brazil, offering courses in health sciences, agricultural sciences, applied social sciences, humanities, and engineering. The convenience sample consisted of first-semester university students of both sexes aged 18 years or older.

### Data collection

Data were collected online, following the Checklist for Reporting Results of Internet E-Surveys (CHERRIES) (Eysenbach, 2004), and took place in February 2021, July 2021, and February 2022. University students were invited to participate in the study during the integration period by being provided with an electronic link to access the research instruments. Upon accessing the electronic link, participants were provided with an informed consent form. After they had read and agreed to the terms, they were directed to the research instruments. Participants who reported suicidal thoughts on item 9 of the PHQ-9 received an automated notification upon completion of the survey. This notification included detailed information about available support services, including the *Centro de Valorização da Vida (CVV)* and the university’s psychological assistance program, which provides both urgent care and ongoing mental health support. Additionally, information about these services was made accessible to all respondents at the end of the questionnaire, regardless of their answers.

### Data collection instruments

Depressive symptoms were measured with the Patient Health Questionnaire-9 (PHQ-9), a standardized instrument developed to identify individuals at increased risk for major depressive episodes and to grade symptom severity. It is used to screen for depressive symptoms in the past two weeks, assessing the presence of nine symptoms according to the fifth edition of the Diagnostic and Statistical Manual of Mental Disorders (DSM-5). Responses are given on a Likert-type scale comprising nine questions categorized into four response options ranging from “not at all” (zero points) to “nearly every day” (3 points) (Santos et al., 2013).

The nine items of the PHQ-9 include the following: PHQ.1 (anhedonia) - “Little interest or pleasure in doing things”; PHQ.2 (depressed mood) - “Feeling down, depressed, or hopeless”; PHQ.3 (sleep) - “Trouble falling asleep, staying asleep, or sleeping too much”; PHQ.4 (energy) - “Feeling tired or having little energy”; PHQ.5 (appetite) - “Poor appetite or overeating”; PHQ.6 (guilt) - “Having negative feelings about yourself, thinking you’re failing, or disappointing yourself or your family”; PHQ.7 (concentration) - “Trouble concentrating on things, such as reading the newspaper or watching television”; and PHQ.8 (motor) - “Moving or speaking so slowly that other people could have noticed. Alternatively, the opposite — being so fidgety or restless that you have been moving around a lot more than usual”; and PHQ.9 (suicide) - “Thoughts that you would be better off dead, or of hurting yourself in some way.”

The PHQ-9 was validated for the general population in Brazil in 2013 and has since demonstrated good psychometric and operational properties, having been validated for both adult and elderly populations (Matias et al., 2016). In this study, the reliability indices of the instrument were 0.902 for Cronbach’s alpha and 0.906 for McDonald’s omega.

The students’ sociodemographic profile was assessed via a questionnaire developed by the researchers, covering the following social profile variables: gender (female; male), age, sexual orientation (heterosexual; sexual minority), relationship status (single; partnered), skin color (white; black; brown), religion (Christian; non-Christian), field of study (health sciences, agricultural sciences, engineering, applied social sciences, and humanities), housing situation (alone; with parents or relatives, with friends), and economic status (minimum wage in Brazil in 2022: BRL 1,212.00, equivalent to USD 214).

### Data analysis and processing

The mean scores of each symptom and the overall PHQ-9 score were compared between heterosexual and sexual minority groups using the Mann-Whitney U test due to the non-normal distribution of the data. For the network analyses, all nine PHQ-9 items were included as nodes. Following current reporting standards for psychological network analyses in cross-sectional data, all preprocessing and analytical decisions were explicitly documented to ensure transparency and reproducibility (Burger et al., 2023).

Initially, we computed summary measures for the PHQ-9 items, which included average scores and their corresponding standard deviations. Subsequently, responses to each item were recoded into binary variables: a score of “0” indicated the absence, and “1” was the presence of a specific depressive symptom. This decision was guided by a conceptual focus on symptom occurrence rather than severity, aiming to model co-occurrence patterns of depressive symptoms within a presence–absence framework. Similar operationalizations have been adopted in prior network studies using the PHQ-9 amid ongoing methodological debates regarding the optimal modeling of ordinal symptom data (Cai et al., 2022). Although this preprocessing step is consistent with the assumptions of the Ising model (Epskamp et al., 2012), it necessarily involves a trade-off between conceptual interpretability (symptom presence vs. absence) and information loss about symptom severity.

To ensure a more interpretable and sparse network, we applied the enhanced Least Absolute Shrinkage and Selection Operator (eLASSO) method, which helps reduce noise by controlling for potential overfitting and excluding weak or non-significant connections between symptoms. The selection of the optimal network structure was guided by the Extended Bayesian Information Criterion (EBIC), which favors models that balance goodness of fit with parsimony. Default tuning parameters implemented in the estimation procedures were retained.

The stability of edge weights was measured by the 95% confidence interval (CI) obtained from 1,000 bootstrap resamples extracted from the study sample, with narrower CIs indicating greater accuracy. The stability of the network was evaluated using the case-dropping procedure to assess the robustness of node centrality after excluding a proportion of the data. To ensure network stability, data exclusion should not result in significant changes in the network. To evaluate the robustness of node centrality estimations, the correlation stability coefficient (CS-C) was applied, following guidelines indicating acceptable values above 0.25 and ideally exceeding 0.5 (Epskamp et al., 2018).

The strength of the relationship between nodes is defined by the edge weights, where a higher value indicates a stronger relationship between the nodes. To examine the most important nodes in the network, centrality was assessed via the degree of centrality or node strength metric. The degree of centrality is evaluated by the sum of the absolute weights of all edges connected to a node. It assesses how central and influential the node is in the network. Owing to instability in bootstrap analyses, betweenness centrality and closeness centrality were not assessed (Dalege et al., 2017).

To compare network structures between heterosexual and sexual minority students, the Network Comparison Test (NCT) was applied using 1,000 permutations to assess differences in global strength and overall network structure (Wang et al., 2020). This approach allowed for assessing whether the overall connectivity (i.e., the cumulative strength of edge associations) differed significantly between the groups. We additionally conducted a simulation-based post-hoc power analysis for the NCT using a Monte Carlo approach. Ising model parameters estimated within each group (edge weights and thresholds) were used to generate binary datasets repeatedly; the NCT was then re-applied to each simulated pair. Power was calculated as the proportion of replications with *p* < 0.05 for network structure and global strength invariance (*R* = 300; 250 permutations), considering *n* = 160, 250, and 350 for the sexual minority group.

Analyses were conducted in R (version 4.0.0) using the packages *bootnet* (Epskamp et al., 2018), *IsingFit* (van Borkulo et al., 2014), *qgraph* (Epskamp et al., 2012), *networktools* (Jones, 2018) and *NetworkComparisonTest* (van Borkulo et al., 2023).

### Ethical considerations

This study followed the recommendations for research protocols involving human subjects and was approved by the Research Ethics Committee (protocol number 4.434.811).

## Results

The sample consisted of 1,271 university students, comprising 850 women (66.9%) and 421 men (33.1%), with an average age of 20.6 years (SD = 5.19). The majority of participants were heterosexual, accounting for 1,111 (87.4%), whereas 160 (12.6%) were sexual minorities. Regarding marital status, 747 (58.8%) participants reported having no partners. In terms of ethnicity, 686 participants (54%) identified as white, and the predominant religion was Christianity, with 1,215 respondents (95.6%). More than half of the participants (710, 55.9%) belonged to the field of health sciences. Concerning economic status, 441 (34.7%) participants reported a monthly income between one and two minimum wages, and 806 (63.4%) lived with their parents or relatives (Table 1).

**Table 1:** Sociodemographic characteristics of heterosexual and sexual minority university students

Variable	*N*	%
Age – mean (SD*)	20.6 (± 5.19)	
Gender		
Female	850	66.9
Male	421	33.1
Sexual orientation		
Heterosexual	1111	87.4
Sexual minority**	160	12.6
Relationship status		
With partner	524	41.2
Without partner	747	58.8
Skin color		
White	686	54
Black	93	7.3
Brown	492	38.7
Religion		
Christian	1215	95.6
Non-Christian	56	4.4
Field of study		
Health sciences	710	55.9
Agricultural sciences	220	17.3
Engineering	104	8.2
Applied social and human sciences	237	18.6
Housing situation		
Alone	381	30
With parents or relatives	806	63.4
With friends	84	6.6
Economic situation***		
Below one minimum wage	218	17.2
1 to 2 minimum wages	441	34.7
3 to 4 minimum wages	251	19.7
Above 4 minimum wages	361	28.4

*SD Standard deviation

**Sexual minority students (gay/lesbian, bisexual, other non-heterosexual identities)

***The minimum wage in Brazil in 2022 was BRL 1,212.00, equivalent to USD 214

Table 2 presents the descriptive analyses of depressive symptoms measured by the PHQ-9, including means, standard deviations, frequency of symptom absence and presence, and the difference between the mean scores of the two groups. After the symptoms were transformed into binary variables, the mean (SD) PHQ-9 score was significantly higher (U = 58416; *p* < 0.001) for the sexual minority group, with a mean score of 5.98 (± 2.58) than for the heterosexual group, which had a mean score of 4.38 (± 2.70). Across all symptoms, the sexual minority group presented significantly higher mean scores than did the heterosexual group (*p* < 0.05).

**Table 2:** Distribution of depressive symptoms (PHQ-9) among heterosexual and sexual minority students

Depressive Symptoms (PHQ-9)	Heterosexuals				Sexual minorities					
	Mean	*SD	Absence %	Presence %	Mean	*SD	Absence %	Presence %	**p	Cohen’s d
PHQ.1	0.58	0.49	41.1	58.9	0.79	0.4	20.6	79.4	< 0.001	0.205
PHQ.2	0.62	0.48	37.6	62.8	0.81	0.39	18.8	81.3	< 0.001	0.184
PHQ.3	0.58	0.49	41.5	58.5	0.75	0.43	24.4	75.6	< 0.001	0.171
PHQ.4	0.76	0.42	23.9	76.1	0.85	0.35	14.4	85.6	0.007	0.094
PHQ.5	0.49	0.50	50.1	49.9	0.67	0.47	32.5	67.5	< 0.001	0.176
PHQ.6	0.46	0.49	53.5	46.5	0.70	0.45	29.4	70.6	< 0.001	0.240
PHQ.7	0.49	0.50	50.2	49.8	0.68	0.46	31.9	68.1	< 0.001	0.183
PHQ.8	0.28	0.44	72.0	28.0	0.46	0.5	53.8	46.3	< 0.001	0.182
PHQ.9	0.08	0.27	91.8	8.2	0.23	0.42	76.3	23.8	< 0.001	0.155

PHQ.1 (anhedonia), PHQ.2 (depressed mood), PHQ.3 (sleep), PHQ.4 (energy), PHQ.5 (appetite), PHQ.6 (guilt), PHQ.7 (concentration), PHQ.8 (motor), PHQ.9 (suicide)

*Standard deviation

**p value from the Mann‒Whitney U test

**Fig. 1:**
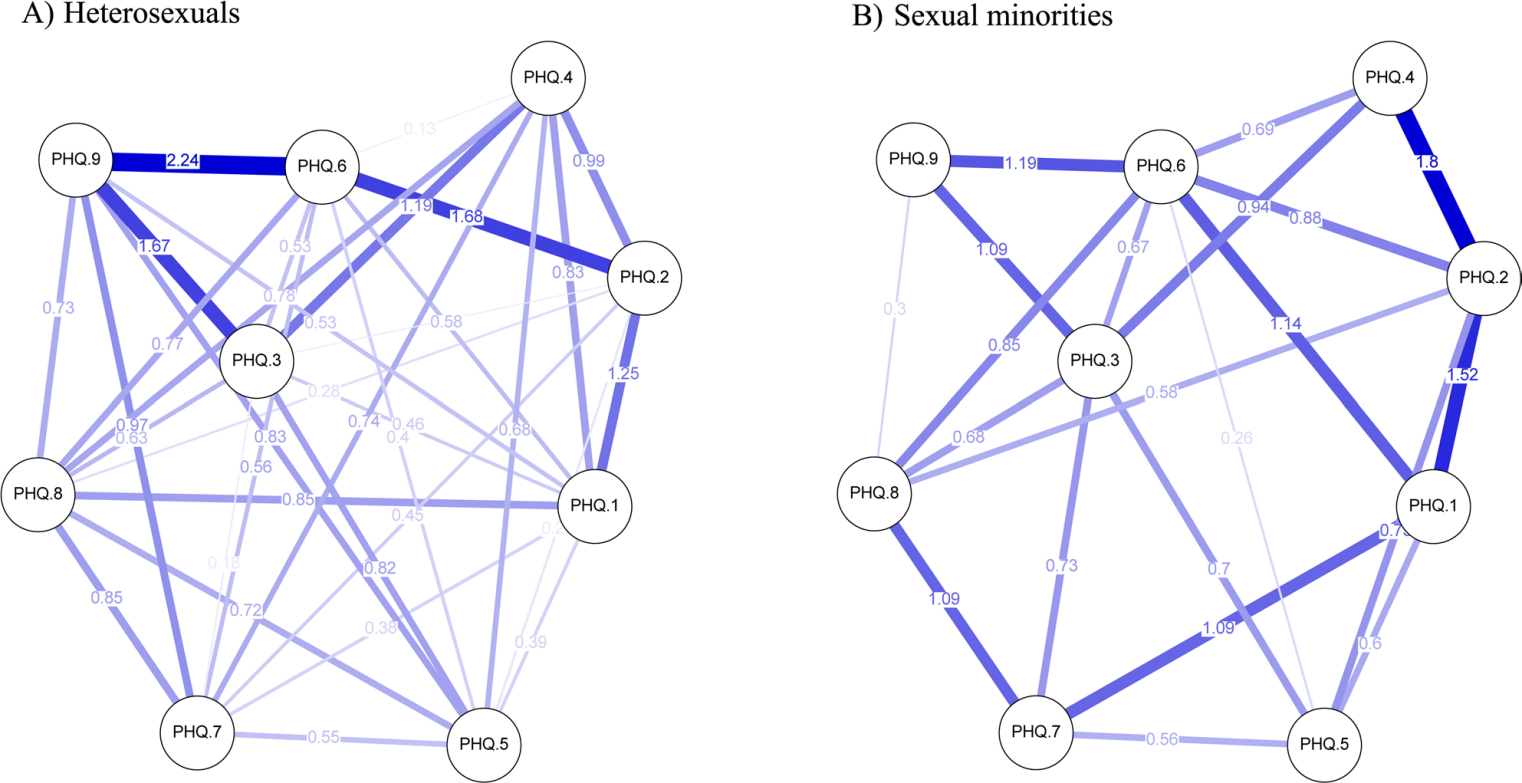
Graphical representation of depressive symptom networks among university students according to sexual orientation. **A** Heterosexual students; **B** Sexual minority students. Note: PHQ.1 (anhedonia); PHQ.2 (depressed mood); PHQ.3 (sleep); PHQ.4 (energy); PHQ.5 (appetite); PHQ.6 (guilt); PHQ.7 (concentration); PHQ.8 (motor); PHQ.9 (suicidal ideation)

Two depressive symptom networks are presented in Fig. 1 for heterosexual and sexual minority groups. In the symptom network of the heterosexual group, the connection between guilt and suicidal ideation (PHQ.6–PHQ.9) had the highest strength, succeeded by the link connecting guilt to depressed mood (PHQ.6–PHQ.2), and then the connection between suicidal ideation and sleep (PHQ.9–PHQ.3). Network analyses of depressive symptoms in the sexual minority student group revealed stronger associations between the edge of depressed mood and energy (PHQ.2–PHQ.4), followed by the edge between depressed mood and anhedonia (PHQ.2–PHQ.1). Weighted adjacency matrix analyses were conducted to examine the numerical interactions between the symptoms (Tables 1 and 2, Supplementary Material).

**Fig. 2:**
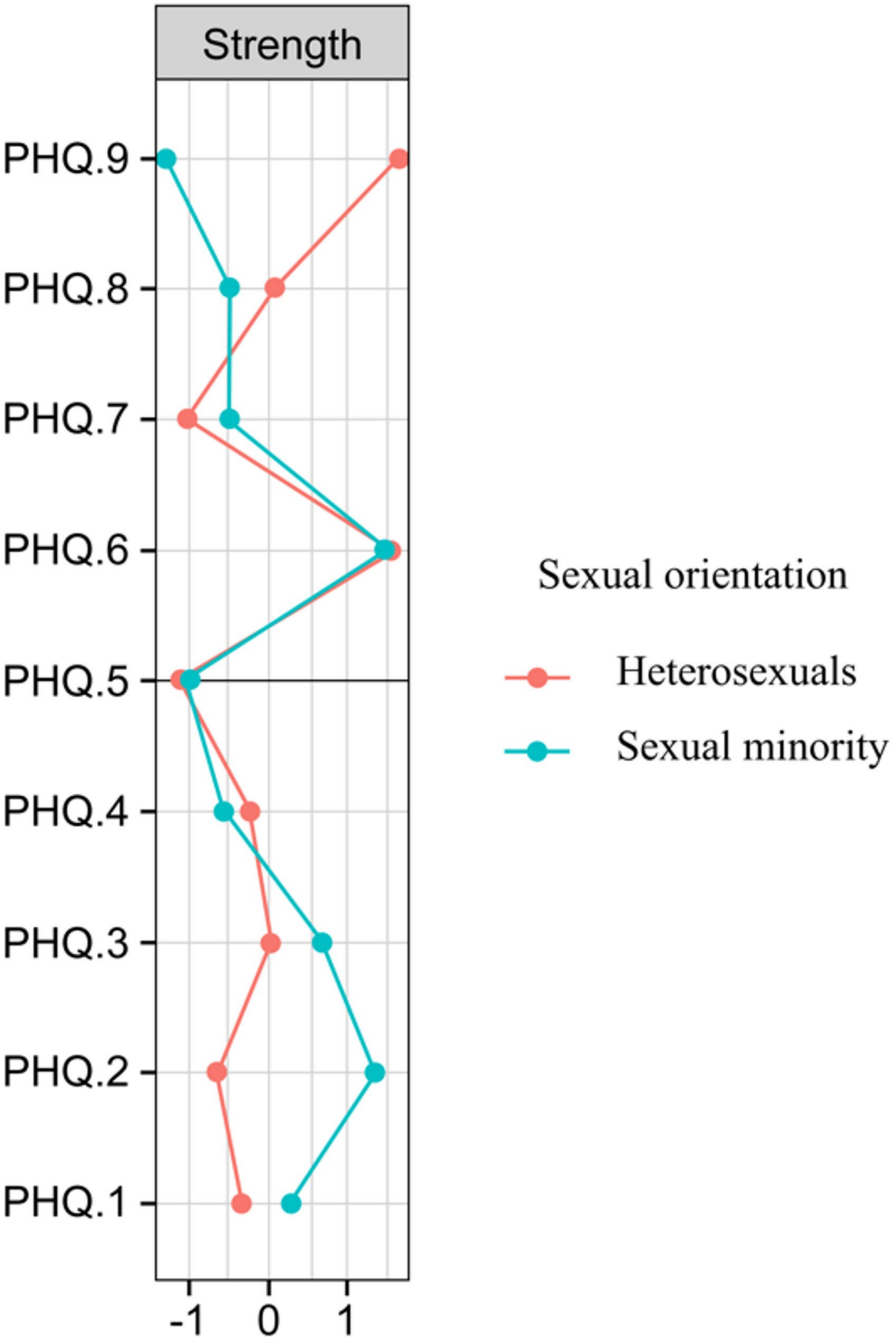
Node centrality (strength) in depressive symptom networks by group. Note: PHQ.1 (anhedonia); PHQ.2 (depressed mood); PHQ.3 (sleep); PHQ.4 (energy); PHQ.5 (appetite); PHQ.6 (guilt); PHQ.7 (concentration); PHQ.8 (motor); PHQ.9 (suicide)

Figure 2 illustrates the node centrality (strength) of all PHQ-9 symptoms in the university student network, differentiated between heterosexual and sexual minority groups. Among the heterosexual students, those with suicidal ideation (PHQ.9) had the highest strength (1.65) in the network. In contrast, for sexual minority students, guilt (PHQ.6) demonstrated the highest strength (1.47) in network analyses. Table 3 in Supplementary Material details the node centrality strength values for each symptom in the network.

**Fig. 3:**
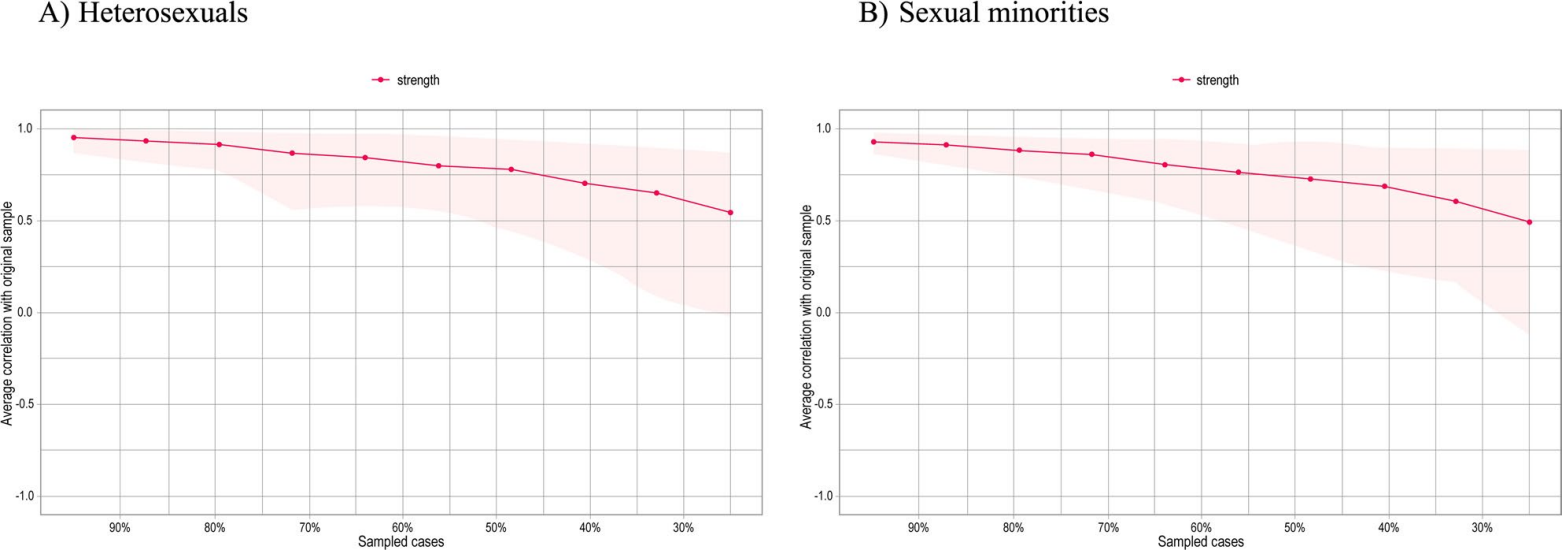
Fig. 3. Centrality stability analysis (strength) based on case-dropping bootstrap. **A** Heterosexual students; **B** Sexual minority students

The bootstrap analyses of edge weights were consistent across both samples, although the sexual minority group exhibited a more skewed distribution (Figs. 1 and 2, Supplementary Material). The stability of both networks was evaluated through a case-dropping bootstrap procedure. The results demonstrated that node strength estimates remained consistent even when a considerable number of observations were systematically removed, as illustrated in Fig. 3. The CS-C values were 0.59 and 0.57 for the heterosexual and sexual minority student groups, respectively. Therefore, it is feasible to interpret symptom strength based on the network analyses performed.

The network comparison test demonstrated the invariance of the network structure and global strength between the two groups. No significant differences were observed in the overall network structure (M = 1.04, *p* = 0.92). Regarding the global strength of each network, no significant difference was found (heterosexual group: 25.01; sexual minority group: 18.11; *p* = 0.65). Simulation-based power estimates indicated limited power to detect differences in overall network structure across group sizes (n_SM = 160: 0.127; n_SM = 250: 0.110; n_SM = 350: 0.337). In contrast, power to detect differences in global strength was moderate for n_SM = 160 (0.693) and high for larger subgroup sizes (n_SM = 250: 0.870; 350: 0.967). These findings suggest that non-significant NCT results—particularly for network structure invariance—should be interpreted cautiously.

## Discussion

This study represents the first known investigation to analyze depressive symptom networks among heterosexual and sexual minority university students. The results demonstrated that sexual minority students experienced significantly greater depressive symptoms than heterosexual students did. As hypothesized, depressive symptom networks showed differences between the groups. Among the heterosexual students, suicidal ideation was the most central symptom in the depression network, followed by guilt. In contrast, among sexual minority students, guilt emerged as the most central symptom, followed by a depressed mood. Differences in network structure across groups may reflect variations in exposure to psychosocial stressors and contextual factors.

An important finding of this study is that suicidal ideation emerged as the central symptom of the depression network among heterosexual students, suggesting that this symptom is more strongly connected with other symptoms and is the most influential symptom in the network. Consistent with the present study, research conducted in different European countries identified suicidal ideation as a central symptom of the general population (Savelieva et al., 2021). However, a systematic review of studies using network analyses to assess depression demonstrated that suicidal ideation generally presents lower centrality indices, suggesting that the relative importance of this symptom may vary across populations and contexts (Malgaroli et al., 2021).

The centrality of suicidal ideation among heterosexual students may also reflect contextual and cultural factors that shape how psychological distress is expressed and organized. University students frequently experience high academic demands, conflicts in the teacher-student relationship, financial pressure, and uncertainty regarding career trajectories, factors that have been consistently associated with increased psychological distress and suicidal ideation (Cecchin et al., 2024; Córdova Olivera et al., 2023). In young adult populations, environments and during periods of heightened uncertainty such as the COVID-19 pandemic, suicidal thoughts may emerge as a cognitively salient and proximal indicator of distress (Matić et al., 2022). Cultural norms that discourage open emotional disclosure or stigmatize mental health difficulties may further contribute to the consolidation of suicidal ideation as a central node within symptom networks, as these norms shape how students perceive and respond to psychological challenges and may influence their willingness to seek help (Cecchin et al., 2024). Importantly, centrality does not imply causality, and this pattern should be interpreted as reflecting how distress becomes structured within specific sociocultural and developmental contexts.

Suicidal behavior, which is the fourth leading cause of death among adolescents and young adults, represents a global public health issue, with suicidal ideation being the first step in this behavior (World Health Organization, 2022). The centrality of suicidal ideation in the depression network of heterosexual university students can be explained by the Interpersonal-Psychological Theory of Suicidal Behavior, which postulates that suicidal ideation arises from high levels of perceived burdensomeness (defined as feeling like a burden on others) and thwarted belongingness (defined as the feeling of not belonging socially). This theory highlights the importance of interpersonal factors in understanding suicidal ideation among university students and highlights the need for psychosocial interventions to address suicidal ideation as it emerges (De Beurs et al., 2019).

Another theory that can explain suicidal ideation as a central symptom among university students is Baumeister’s Escape Theory (Baumeister, 1990). Dealing with the new reality of the university environment, along with academic and social pressure, may lead to unmet expectations, resulting in negative self-assessment. When university students struggle to cope with academic and social demands, negative self-assessment can lead to suicidal ideation as a way to escape negative emotions. In this context, educational institutions need to create self-efficacy strategies to help reduce negative self-assessment.

In the network of depressive symptoms, guilt emerged as the most central node among sexual minority students, while in the heterosexual student network it ranked as the second most influential symptom. This finding is consistent with network analyses conducted on adolescents (Cai et al., 2022) and elderly individuals (Tao et al., 2023), which also identified guilt as a central symptom. Guilt, understood as a negative self-assessment of one’s behavior, is often linked to self-blame, especially when one’s sexual identity and orientation are seen as sources of suffering. These perceptions can have severe adverse impacts on students’ mental health and overall well-being. A study conducted with sexual minority adolescents revealed that family rejection is associated with greater feelings of guilt (Mereish et al., 2021). Sexual minority youth frequently experience discrimination and prejudice because of their sexual orientation. These experiences of victimization, including bullying, also occur in university settings and can lead to feelings of guilt (Li et al., 2023).

The Minority Stress Theory has been widely applied to explain mental health disparities between sexual minority and heterosexual individuals. This framework suggests that sexual minority individuals are exposed to distal stressors, such as discrimination, prejudice, and victimization, while also encountering proximal stressors, like expectations of rejection, concealment of identity, and internalized stigma. Together, these stressors increase vulnerability to adverse mental health outcomes (Meyer, 2003). In this context, guilt may be especially central in the depressive symptom network among sexual minority students, likely reflecting the internalization of stigma-related experiences (Caculidis-Tudor et al., 2023). Recent evidence suggests that emotions involving negative self-evaluation—shame and guilt—play a key role in linking minority stress to psychological distress (Carlisle et al., 2026). Building on these ideas, the Psychological Mediation Framework expands the Minority Stress Theory by proposing that general psychological processes mediate the relationship between minority stress and mental health outcomes (Hatzenbuehler, 2009). Within this framework, exposure to stigma-related stress may trigger maladaptive cognitive and emotional processes, such as rumination and negative self-evaluation, which contribute to depressive symptoms. Therefore, the prominent position of guilt in the depressive symptom network observed in the present study may represent a manifestation of internalized stigma and self-critical cognitive processes that reinforce and sustain depressive symptomatology.

Understanding the central symptoms of depression networks is important because highly connected symptoms may play a prominent role in the overall organization of psychopathological systems. The network theory of psychopathology emphasizes that psychological states and symptoms mutually influence each other, and this dynamic plays an important role in the subsequent development of psychopathology (Wichers et al., 2021). From this perspective, identifying central symptoms may provide useful hypotheses regarding potential intervention targets.

In this study, the strongest associations in the heterosexual student network were observed between guilt and suicidal ideation (PHQ.6 and PHQ.9). There is a bidirectional relationship between suicide and psychiatric symptoms, both of which can result from suicide. Depression is recognized as an important risk factor for suicide among young people as it intensifies feelings of hopelessness and guilt, increasing the likelihood of suicidal ideation (Cai et al., 2023).

In contrast, in the sexual minority group, the strongest associations were found between depressed mood and lack of energy (PHQ.2 and PHQ.4). This pattern may be partially explained by proximal stressors described in the Minority Stress Theory, particularly expectations of rejection and identity concealment. Sexual minority individuals frequently engage in continuous self-monitoring in social interactions in order to avoid revealing signs of their sexual orientation and to prevent potential experiences of stigma or discrimination. This constant state of vigilance may be cognitively and emotionally exhausting, contributing to persistent feelings of fatigue and depleted psychological resources (Pachankis et al., 2020). Over time, the interaction between these minority stressors and concealment strategies may increase vulnerability to depressive symptoms, particularly lack of energy and depressed mood. Additionally, university students often face academic stress, which can further exacerbate symptoms of low energy (Pérez-Jorge et al., 2025). These findings may help inform interventions aimed at improving coping with minority stress and managing academic challenges among sexual minority university students. Depressed mood is also widely recognized as a hallmark symptom of depression, and its centrality aligns with the theoretical conceptualization of the disorder (Xie et al., 2022).

The results of this study showed that sexual minority students had significantly greater levels of depressive symptoms than heterosexual students. Scientific evidence has demonstrated a mental health disparity between sexual minorities and heterosexuals, as indicated by significantly higher rates of depression among sexual minorities (Camargo Júnior et al., 2023).

It is important to consider that data collection occurred during the COVID-19 pandemic, a period associated with substantial psychological distress globally (Chen et al. 2025a, 2025b). Brazil was especially affected, with one of the highest mortality rates (Szwarcwald et al., 2022). Social distancing, remote learning, and reduced social interaction likely influenced students’ emotional experiences and symptom reporting. Consequently, some observed depressive symptoms, such as feelings of guilt and psychological exhaustion, may partially reflect the broader psychosocial impact of the pandemic rather than solely academic or minority stressors.

### Limitations

Despite advances in understanding the depressive symptom networks of heterosexual and sexual minority students, the results of this study should be interpreted in the light of several limitations. This study employed a cross-sectional approach and was conducted during the COVID-19 pandemic; therefore, further studies are needed post-pandemic, as well as dynamic network analyses using a longitudinal approach. The sample of students belonging to sexual minorities was smaller than that of heterosexual students. Bootstrap analyses suggested acceptable accuracy and stability of several network estimates; however, the smaller subgroup size may have limited sensitivity for between-group comparisons. A methodological limitation of this study is the dichotomization of PHQ-9 items before network estimation which may reduce information and affect network recovery and parameter estimation (MacCallum et al., 2002; Sekulovski et al., 2025). Therefore, findings should be interpreted with caution, and future studies are encouraged to apply ordinal network approaches.

Psychological symptom networks estimated from cross-sectional data represent statistical patterns of conditional association and should not be interpreted as direct evidence of causal mechanisms linking symptoms. Recent critiques highlight that symptom networks may show limited replicability across samples, and that there are inferential gaps between observed statistical networks and the theoretical models often used to interpret them (McNally, 2021; Forbes et al., 2017; Fried, 2020). Although our within-group network metrics showed acceptable stability (CS-C > 0.50 for strength centrality), simulation-based post-hoc analyses indicated limited power to detect between-group differences in overall network structure. Specifically, power for network structure invariance remained low across scenarios, suggesting that the absence of significant structural differences in the NCT should be interpreted cautiously and may reflect Type II error. In contrast, power to detect differences in global strength was moderate for the observed sexual minority sample size and increased substantially for larger subgroup sizes. Future studies should aim to recruit larger sexual minority samples to improve the sensitivity of structural network comparisons, while our current findings primarily inform within-group symptom centrality patterns.

### Implication for practice

The findings may have practical implications for health and educational settings by informing screening priorities and generating clinically plausible hypotheses about symptom constellations in different student groups. However, because cross-sectional symptom networks reflect statistical associations rather than confirmed causal processes, intervention targets should not be selected solely based on centrality indices (Fried, 2020). Instead, the present results can support assessment, monitoring, and referral pathways, while preventive and therapeutic strategies should remain grounded in established clinical evidence and theory.

For heterosexual students, prevention and care strategies may prioritize early identification of suicidal ideation through regular screening in health services and educational institutions, alongside clear referral pathways and timely mental health support. For sexual minority students, care may benefit from addressing social isolation and guilt-related experiences. Cognitive-behavioral approaches may be considered to strengthen self-efficacy and reduce dysfunctional automatic thoughts associated with guilt, while structured support groups may provide safe contexts to discuss and manage guilt-related experiences. Low-intensity strategies, such as mindfulness-based practices, may also be offered to both heterosexual and sexual minority students. Mindfulness-based meditation has shown promising results in improving mental health and may help reduce exhaustion and stress associated with the academic environment (Gherardi-Donato et al., 2023).

## Conclusion

Psychopathology network analyses revealed that different depressive symptoms played distinct central roles among university student groups. Among heterosexual students, suicidal ideation was the most central and influential symptom, whereas guilt had a greater centrality among sexual minorities.

While these results may inform assessment and monitoring priorities, centrality in cross-sectional networks is descriptive and does not establish causal mechanisms or definitive treatment targets. Replication in larger sexual minority samples and longitudinal or intervention studies are needed to evaluate whether changes in these symptoms precede or contribute to broader symptom improvement.

## Supplementary Information

Supplementary Material 1.

## Data Availability

No datasets were generated or analysed during the current study.

## References

[CR1] Argyriou, A., Goldsmith, K. A., & Rimes, K. A. (2021). Mediators of the Disparities in Depression Between Sexual Minority and Heterosexual Individuals: A Systematic Review. *Archives of Sexual Behavior*, *50*, 925–959. 10.1007/s10508-020-01862-0 33689086 10.1007/s10508-020-01862-0 PMC8035121

[CR2] Balloo, K., Hosein, A., Byrom, N., & Essau, C. A. (2022). Differences in mental health inequalities based on university attendance: Intersectional multilevel analyses of individual heterogeneity and discriminatory accuracy. *SSM - Population Health*, *19*, 101149. 10.1016/j.ssmph.2022.101149 35800663 10.1016/j.ssmph.2022.101149 PMC9253404

[CR3] Baumeister, R. F. (1990). Suicide as escape from self. *Psychological Review*, *97*, 90–113. 10.1037/0033-295x.97.1.90 2408091 10.1037/0033-295x.97.1.90

[CR4] Borsboom, D. (2017). A network theory of mental disorders. *World Psychiatry: official journal of the World Psychiatric Association (WPA)*, *16*(1), 5–13. 10.1002/wps.20375 28127906 10.1002/wps.20375 PMC5269502

[CR5] Borsboom, D., & Cramer, A. O. J. (2013). Network Analysis: An Integrative Approach to the Structure of Psychopathology. *Annual Review Clinical Psychology*, *9*, 91–121. 10.1146/annurev-clinpsy-050212-185608 10.1146/annurev-clinpsy-050212-185608 23537483

[CR6] Brooks, J. R., Makhubela, M., Madubata, I., & Walker, R. L. (2025). Network Analysis of Depression Symptoms in United States and South African University Students. *International Perspectives in Psychology*, *14*, 18–29. 10.1027/2157-3891/a000116

[CR7] Burger, J., Isvoranu, A. M., Lunansky, G., Haslbeck, J. M. B., Epskamp, S., Hoekstra, R. H. A., Fried, E. I., Borsboom, D., & Blanken, T. F. (2023). Reporting standards for psychological network analyses in cross-sectional data. *Psychological Methods*, *28*(4), 806–824. 10.1037/met0000471 35404629 10.1037/met0000471

[CR8] Caculidis-Tudor, D., Bică, A., Ianole-Călin, R., & Podina, I. R. (2023). The less I get, the more I punish: A moderated-mediation model of rejection sensitivity and guilt in depression. *Current Psychology*, *42*, 3567–3579. 10.1007/s12144-021-01625-7

[CR9] Cai, H., Bai, W., Liu, H., Chen, X., Qi, H., Liu, R., Cheung, T., Su, Z., Lin, J., Tang, Y., Jackson, T., Zhang, Q., & Xiang, Y. T. (2022). Network analysis of depressive and anxiety symptoms in adolescents during the later stage of the COVID-19 pandemic. *Translational Psychiatry*, *12*, 1–8. 10.1038/s41398-022-01838-9 35273161 10.1038/s41398-022-01838-9 PMC8907388

[CR10] Cai, H., Chow, I. H. I., Lei, S. M., Lok, G. K. I., Su, Z., Cheung, T., Peshkovskaya, A., Tang, Y. L., Jackson, T., Ungvari, G. S., Zhang, L., & Xiang, Y. T. (2023). Inter-relationships of depressive and anxiety symptoms with suicidality among adolescents: A network perspective. *Journal of Affective Disorders*, *324*, 480–488. 10.1016/j.jad.2022.12.093 36584712 10.1016/j.jad.2022.12.093

[CR11] Camargo Júnior, E. B., Noivo, I. S., Gouvea, T. C. C., Fernandes, M. N., & de Gherardi-Donato, F. (2023). E.C. da S. Depression and Substance Use Among Brazilian University Students During the COVID-19 Pandemic. *Journal of Psychoactive Drugs*, 1–10. 10.1080/02791072.2023.2244499 10.1080/02791072.2023.2244499 37551709

[CR13] Carlisle, N. A., Miller, P., Pavela, G., Montgomery, A. E., Miller, G. H., Heath, S. L., Hendricks, P. S., & MacCarthy, S. (2026). The price of shame: A scoping review examining the effects of shame on sexual and gender minority populations in the United States. *SSM - Mental Health*, *9*, 100595. 10.1016/j.ssmmh.2026.100595

[CR14] Cecchin, H. F. G., da Costa, H. E. R., Pacheco, G. R., de Valencia, G. B., & Murta, S. G. (2024). Risk Factors for Suicidal Ideation in Brazilian University Students: A Mixed Methods Study. *Trends in Psychology*. 10.1007/s43076-024-00402-2

[CR12] Chen, Z., Bai, J., Hu, Y., & Wang, Y. (2025a). Social Anxiety and Its Comorbidity with Depression in Medical Students: A Network Analysis. *Stress and Health*, *41*, e70045. 10.1002/smi.70045 40298284 10.1002/smi.70045

[CR15] Chen, M., Miao, J., Chen, C., Qu, R., Zhou, W., Qi, J., Cao, K., Wu, X., Wang, Y., Yang, Y., Zhou, J., Yan, R., Dong, N., Zhu, C., & Yang, S. (2025b). The impact of the COVID-19 pandemic on the global burden of mental disorders: A counterfactual modeling study from 1990 to 2021. *Translational Psychiatry*, *15*(1), 493. 10.1038/s41398-025-03697-6 41271619 10.1038/s41398-025-03697-6 PMC12639033

[CR16] Cheung, T., Jin, Y., Lam, S., Su, Z., Hall, B. J., & Xiang, Y. T. (2021). Network analysis of depressive symptoms in Hong Kong residents during the COVID-19 pandemic. *Translation Psychiatry*, *11*, 460. 10.1038/s41398-021-01543-z 10.1038/s41398-021-01543-z PMC8419676 34489416

[CR17] Córdova Olivera, P., Gasser Gordillo, P., Naranjo Mejía, H., La Fuente Taborga, I., Grajeda Chacón, A., & Sanjinés Unzueta, A. (2023). Academic stress as a predictor of mental health in university students. *Cogent Education*, *10*(2). 10.1080/2331186X.2023.2232686

[CR18] Dalege, J., Borsboom, D., van Harreveld, F., & van der Maas, H. L. J. (2017). Network Analysis on Attitudes: A Brief Tutorial. *Social Psychological and Personality Science*, *8*, 528–537. 10.1177/1948550617709827 28919944 10.1177/1948550617709827 PMC5582642

[CR19] De Beurs, D., Fried, E. I., Wetherall, K., Cleare, S., O’ Connor, D. B., Ferguson, E., O’Carroll, R. E., & O’ Connor, R. C. (2019). Exploring the psychology of suicidal ideation: A theory driven network analysis. *Behaviour Research and Therapy*, *120*, 103419. 10.1016/j.brat.2019.103419 31238299 10.1016/j.brat.2019.103419

[CR21] Epskamp, S., Cramer, A. O. J., Waldorp, L. J., Schmittmann, V. D., & Borsboom, D. (2012). qgraph: Network Visualizations of Relationships in Psychometric Data. *Journal of Statistical Software*, *48*, 1–18. 10.18637/jss.v048.i04

[CR20] Epskamp, S., Borsboom, D., & Fried, E. I. (2018). Estimating psychological networks and their accuracy: A tutorial paper. *Behavior Research Methods*, *50*, 195–212. 10.3758/s13428-017-0862-1 28342071 10.3758/s13428-017-0862-1 PMC5809547

[CR22] Eysenbach, G. (2004). Improving the quality of Web surveys: the Checklist for Reporting Results of Internet E-Surveys (CHERRIES). *Journal of Medical Internet Research*, *6*(3), e34. 10.2196/jmir.6.3.e34 15471760 10.2196/jmir.6.3.e34 PMC1550605

[CR23] Filatova, E. V., Shadrina, M. I., & Slominsky, P. A. (2021). Major Depression: One Brain, One Disease, One Set of Intertwined Processes. *Cells*, *10*, 1283. 10.3390/cells10061283 34064233 10.3390/cells10061283 PMC8224372

[CR24] Forbes, M. K., Wright, A. G. C., Markon, K. E., & Krueger, R. F. (2017). Evidence that psychopathology symptom networks have limited replicability. *Journal of Abnormal Psychology*, *126*(7), 969–988. 10.1037/abn0000276 29106281 10.1037/abn0000276 PMC5749927

[CR25] Fried, E. I. (2015). Problematic assumptions have slowed down depression research: why symptoms, not syndromes are the way forward. *Frontiers in Psychology*. 10.3389/fpsyg.2015.00309. 6. 25852621 10.3389/fpsyg.2015.00309 PMC4369644

[CR26] Fried, E. I. (2020). Lack of theory building and testing impedes progress in the factor and network literature. *Psychological Inquiry*, *31*(4), 271–288. 10.1080/1047840X.2020.1853461

[CR27] Frost, D. M., & Meyer, I. H. (2023). Minority stress theory: Application, critique, and continued relevance. *Current Opinion in Psychology*, *51*, 101579. 10.1016/j.copsyc.2023.101579 37270877 10.1016/j.copsyc.2023.101579 PMC10712335

[CR28] Gauld, C., Baillet, E., Micoulaud-Franchi, J. A., Kervran, C., Serre, F., & Auriacombe, M. (2023). The centrality of craving in network analysis of five substance use disorders. *Drug and Alcohol Dependence*, *245*, 109828. 10.1016/j.drugalcdep.2023.109828 36868091 10.1016/j.drugalcdep.2023.109828

[CR29] Gherardi-Donato, E. C. S., Gimenez, L. B. H., Fernandes, M. N., de Lacchini, F., Camargo, R., Júnior, E. B., Díaz-Serrano, K. V., Melchior, M., Pérez, R. G., Riquelme-Galindo, J., & Reisdorfer, E. (2023). Mindfulness Practice Reduces Hair Cortisol, Anxiety and Perceived Stress in University Workers: Randomized Clinical Trial. *Healthcare*, *11*, 2875. 10.3390/healthcare11212875 37958019 10.3390/healthcare11212875 PMC10648523

[CR30] Gomes, N. L., & Lopes, C. S. (2023). Sexual Orientation Disparities in Depression and Substance Use Among Adults: Results from the Brazilian National Health Survey 2019. *LGBT Health*, *10*, 363–371. 10.1089/lgbt.2022.0193 36809196 10.1089/lgbt.2022.0193

[CR31] Hatzenbuehler, M. L. (2009). How does sexual minority stigma get under the skin? A psychological mediation framework. *Psychological Bulletin*, *135*(5), 707–730. 10.1037/a0016441 19702379 10.1037/a0016441 PMC2789474

[CR32] Helminen, E. C., Scheer, J. R., Ash, T. L., Haik, A. K., & Felver, J. C. (2023). Discrimination, Depression, and Anxiety Among Sexual Minority and Heterosexual Young Adults: The Role of Self-Compassion. *LGBT Health*, *10*, 315. 10.1089/lgbt.2022.0079 36656549 10.1089/lgbt.2022.0079 PMC10329156

[CR33] Institute of Health Metrics and Evaluation (2021). Global Burden of Disease (GBD) [Document]. Institute for Health Metrics and Evaluation. URL https://vizhub.healthdata.org/gbd-results. Accessed 10 June 2024.

[CR34] Jones, P. J. (2018). *networktools: Tools for identifying important nodes in networks*. R package version 1.2.3. https://CRAN.R-project.org/package=networktools

[CR35] Li, J., Jin, Y., Xu, S., Wilson, A., Chen, C., Luo, X., Liu, Y., Ling, X., Sun, X., & Wang, Y. (2023). Effects of Bullying on Anxiety, Depression, and Posttraumatic Stress Disorder Among Sexual Minority Youths: Network Analysis. *JMIR Public Health Surveill*, *9*, e47233. 10.2196/47233 37910159 10.2196/47233 PMC10652196

[CR36] Li, R., Shi, C., Yang, W., Liu, X., & Ren, Z. (2025). Network Analysis of Depressive Symptoms in Chinese Sexual Minority Women During the COVID-19 Pandemic: An Intra-Group Perspective. *Journal of Homosexuality*, *72*, 914–930. 10.1080/00918369.2024.2359950 38833635 10.1080/00918369.2024.2359950

[CR37] Liu, S., Ren, H., Li, Y., Liu, Y., Fu, S., & Han, Z. R. (2025). Gender Difference in the Onset of Adolescent Depressive Symptoms: A Cross-Lagged Panel Network Analysis. *Research on Child and Adolescent Psychopathology*, *53*, 113–123. 10.1007/s10802-024-01235-4 39215790 10.1007/s10802-024-01235-4

[CR38] MacCallum, R. C., Zhang, S., Preacher, K. J., & Rucker, D. D. (2002). On the practice of dichotomization of quantitative variables. *Psychological Methods*, *7*(1), 19–40. 10.1037/1082-989x.7.1.19 11928888 10.1037/1082-989x.7.1.19

[CR39] Malgaroli, M., Calderon, A., & Bonanno, G. A. (2021). Networks of major depressive disorder: A systematic review. *Clinical Psychology Review*, *85*, 102000. 10.1016/j.cpr.2021.102000 33721606 10.1016/j.cpr.2021.102000

[CR40] Matias, A. G. C., Fonsêca, M. A., Gomes, M. L., & de Matos, F., M.A.A (2016). Indicators of depression in elderly and different screening methods. *Einstein*, *14*, 6. 10.1590/S1679-45082016AO3447 27074227 10.1590/S1679-45082016AO3447 PMC4872910

[CR41] Matić, T., Pregelj, P., Sadikov, A., & Prelog, P. R. (2022). Depression, Anxiety, Stress, and Suicidality Levels in Young Adults Increased Two Years into the COVID-19 Pandemic. *International Journal of Environmental Research and Public Health*, *20*(1). 10.3390/ijerph20010339 10.3390/ijerph20010339 PMC9819448 36612666

[CR42] McNally, R. J. (2021). Network analysis of psychopathology: Controversies and challenges. *Annual Review of Clinical Psychology*, *17*, 31–53. 10.1146/annurev-clinpsy-081219-092850 33228401 10.1146/annurev-clinpsy-081219-092850

[CR43] Mereish, E. H., Cox, D. J., Harris, J. C., Anderson, Q. R., & Hawthorne, D. J. (2021). Emerging Ideas. Familial Influences, Shame, Guilt, and Depression Among Sexual Minority Adolescents. *Family Relations*, *70*, 1546–1555. 10.1111/fare.12514

[CR44] Meyer, I. H. (2003). Prejudice, Social Stress, and Mental Health in Lesbian, Gay, and Bisexual Populations: Conceptual Issues and Research Evidence. *Psychological Bulletin*, *129*, 674–697. 10.1037/0033-2909.129.5.674 12956539 10.1037/0033-2909.129.5.674 PMC2072932

[CR45] Pachankis, J. E., Jackson, S. D., Fetzner, B. K., Mahon, C. P., & Bränström, R. (2020). Sexual Orientation Concealment and Mental Health: A Conceptual and Meta-Analytic Review. *Psychological Bulletin*, *146*(10), 831. 10.1037/bul0000271 32700941 10.1037/bul0000271 PMC8011357

[CR46] Pachankis, J. E., Hatzenbuehler, M. L., Klein, D. N., & Bränström, R. (2024). The Role of Shame in the Sexual-Orientation Disparity in Mental Health: A Prospective Population-Based Study of Multimodal Emotional Reactions to Stigma. *Clinical Psychological Science: Journal of the Association for Psychological Science*, *12*(3), 486–504. 10.1177/21677026231177714 10.1177/21677026231177714 PMC11210704 38938414

[CR47] Pérez-Jorge, D., Boutaba-Alehyan, M., González-Contreras, A. I., & Pérez-Pérez, I. (2025). Examining the effects of academic stress on student well-being in higher education. *Humanities and Social Sciences Communications*, *12*(1), 449. 10.1057/s41599-025-04698-y

[CR48] Santos, I. S., Tavares, B. F., Munhoz, T. N., de Almeida, L. S. P., Silva, N. T. B., da, Tams, B. D., Patella, A. M., & Matijasevich, A. (2013). Sensibilidade e especificidade do Patient Health Questionnaire-9 (PHQ-9) entre adultos da população geral. *Cadernos de Saúde Pública*, *29*, 1533–1543. 10.1590/0102-311X00144612 24005919 10.1590/0102-311x00144612

[CR49] Savelieva, K., Komulainen, K., Elovainio, M., & Jokela, M. (2021). Longitudinal associations between specific symptoms of depression: Network analysis in a prospective cohort study. *Journal of Affective Disorders*, *278*, 99–106. 10.1016/j.jad.2020.09.024 32956966 10.1016/j.jad.2020.09.024

[CR50] Sekulovski, N., Blanken, T. F., Haslbeck, J. M. B., & Marsman, M. (2025). The impact of dichotomization on network recovery. *Behavior Research Methods*, *57*, 342. 10.3758/s13428-025-02861-6 41219655 10.3758/s13428-025-02861-6 PMC12605567

[CR51] Sheldon, E., Simmonds-Buckley, M., Bone, C., Mascarenhas, T., Chan, N., Wincott, M., Gleeson, H., Sow, K., Hind, D., & Barkham, M. (2021). Prevalence and risk factors for mental health problems in university undergraduate students: A systematic review with meta-analysis. *Journal of Affective Disorders*, *287*, 282–292. 10.1016/j.jad.2021.03.054 33812241 10.1016/j.jad.2021.03.054

[CR52] Szwarcwald, C. L., Boccolini, C. S., Almeida, W. S., Filho, S., A. M., & Malta, D. C. (2022). COVID-19 mortality in Brazil, 2020-21: consequences of the pandemic inadequate management. *Archives of Public Health*, *80*(255). 10.1186/s13690-022-01012-z 10.1186/s13690-022-01012-z PMC9762984 36536434

[CR53] Tao, Y., Hou, W., Niu, H., Ma, Z., Zheng, Z., Wang, S., Liu, X., & Zhang, L. (2023). Comparing the centrality symptoms of major depressive disorder samples across junior high school students, senior high school students, college students and elderly adults during city lockdown of COVID-19 pandemic-A network analysis. *Journal of Affective Disorders*, *324*, 190–198. 10.1016/j.jad.2022.12.120 36586620 10.1016/j.jad.2022.12.120 PMC9797224

[CR54] Terra, T., Schafer, J. L., Pan, P. M., Costa, A. B., Caye, A., Gadelha, A., Miguel, E. C., Bressan, R. A., Rohde, L. A., & Salum, G. A. (2022). Mental health conditions in Lesbian, Gay, Bisexual, Transgender, Queer and Asexual youth in Brazil: A call for action. *Journal of Affective Disorders*, *298*, 190–193. 10.1016/j.jad.2021.10.108 34715179 10.1016/j.jad.2021.10.108

[CR55] van Borkulo, C. D., Epskamp, S., Millner, A. J., Waldorp, L. J., & Borsboom, D. (2014). A new method for constructing networks from binary data. *Scientific Reports*, *4*, 5918. 10.1038/srep05918 25082149 10.1038/srep05918 PMC4118196

[CR56] van Borkulo, C. D., van Bork, R., Boschloo, L., Kossakowski, J. J., Tio, P., Schoevers, R. A., Borsboom, D., & Waldorp, L. J. (2023). Comparing network structures on three aspects: A permutation test. *Psychological Methods*, *28*(6), 1273–1285. 10.1037/met0000476 35404628 10.1037/met0000476

[CR57] Wang, Y., Hu, Z., Feng, Y., Wilson, A., & Chen, R. (2020). Changes in network centrality of psychopathology symptoms between the COVID-19 outbreak and after peak. *Molecular Psychiatry*, *25*, 3140–3149. 10.1038/s41380-020-00881-6 32929212 10.1038/s41380-020-00881-6 PMC7488637

[CR58] Wichers, M., Riese, H., Hodges, T. M., Snippe, E., & Bos, F. M. (2021). A Narrative Review of Network Studies in Depression: What Different Methodological Approaches Tell Us About Depression. *Frontiers in Psychiatry*, *12*, 719490. 10.3389/fpsyt.2021.719490 34777038 10.3389/fpsyt.2021.719490 PMC8581034

[CR59] Wittgens, C., Fischer, M. M., Buspavanich, P., Theobald, S., Schweizer, K., & Trautmann, S. (2022). Mental health in people with minority sexual orientations: A meta-analysis of population-based studies. *Acta Psychiatrica Scandinavica*, *145*, 357–372. 10.1111/acps.13405 35090051 10.1111/acps.13405

[CR60] World Health Organization, W. (2022). *World mental health report: Transforming mental health for all*. from https://www.who.int/publications/i/item/9789240049338. Retrieved 10 June 2024

[CR61] Xie, T., Wen, J., Liu, X., Wang, J., & Poppen, P. J. (2022). Utilizing network analysis to understand the structure of depression in Chinese adolescents: Replication with three depression scales. *Current Psychology*, 1–12. 10.1007/s12144-022-03201-z 10.1007/s12144-022-03201-z PMC9157480 35669214

[CR62] Zhao, Y., Qu, D., Chen, S., & Chi, X. (2023). Network analysis of internet addiction and depression among Chinese college students during the COVID-19 pandemic: A longitudinal study. *Computers in Human Behavior*, *138*, 107424. 10.1016/j.chb.2022.107424 35945974 10.1016/j.chb.2022.107424 PMC9352366

