# Supplementary material for: Comparison of depressive symptoms between heterosexual and sexual minority university students: network analysis
Source: Psicol Reflex Crit. 2026 Apr 6;39:17. doi: 10.1186/s41155-026-00388-z (PMC13181111; doi:10.1186/s41155-026-00388-z)
Supplement: Supplementary file 1 — Supplementary Material 1. [file 41155_2026_388_MOESM1_ESM.docx]

**SUPPLEMENTARY MATERIAL**

**Table 1 -** Weighting matrix of network analysis for heterosexual students.

|  | | **Heterosexual** | | | | | | | | | | | | | | | | | |  |
| --- | --- | --- | --- | --- | --- | --- | --- | --- | --- | --- | --- | --- | --- | --- | --- | --- | --- | --- | --- | --- |
| **Variável** | | **PHQ.1** | | **PHQ.2** | | **PHQ.3** | | **PHQ.4** | | **PHQ.5** | | **PHQ.6** | | **PHQ.7** | | **PHQ.8** | | **PHQ.9** | |  |
| PHQ.1 |  | 0.000 |  | 1.252 |  | 0.456 |  | 0.834 |  | 0.387 |  | 0.583 |  | 0.378 |  | 0.847 |  | 0.531 |  |  |
| PHQ.2 |  | 1.252 |  | 0.000 |  | 0.100 |  | 0.990 |  | 0.249 |  | 1.676 |  | 0.449 |  | 0.283 |  | 0.000 |  |  |
| PHQ.3 |  | 0.456 |  | 0.100 |  | 0.000 |  | 1.194 |  | 0.824 |  | 0.534 |  | 0.184 |  | 0.634 |  | 1.672 |  |  |
| PHQ.4 |  | 0.834 |  | 0.990 |  | 1.194 |  | 0.000 |  | 0.678 |  | 0.125 |  | 0.739 |  | 0.784 |  | 0.000 |  |  |
| PHQ.5 |  | 0.387 |  | 0.249 |  | 0.824 |  | 0.678 |  | 0.000 |  | 0.397 |  | 0.546 |  | 0.722 |  | 0.826 |  |  |
| PHQ.6 |  | 0.583 |  | 1.676 |  | 0.534 |  | 0.125 |  | 0.397 |  | 0.000 |  | 0.563 |  | 0.771 |  | 2.242 |  |  |
| PHQ.7 |  | 0.378 |  | 0.449 |  | 0.184 |  | 0.739 |  | 0.546 |  | 0.563 |  | 0.000 |  | 0.850 |  | 0.972 |  |  |
| PHQ.8 |  | 0.847 |  | 0.283 |  | 0.634 |  | 0.784 |  | 0.722 |  | 0.771 |  | 0.850 |  | 0.000 |  | 0.733 |  |  |
| PHQ.9 |  | 0.531 |  | 0.000 |  | 1.672 |  | 0.000 |  | 0.826 |  | 2.242 |  | 0.972 |  | 0.733 |  | 0.000 |  |  |

**Table 2 -** Weighting matrix of network analysis for sexual minority students.

|  | **Sexual minority** | | | | | | | | | | | | | | | | |
| --- | --- | --- | --- | --- | --- | --- | --- | --- | --- | --- | --- | --- | --- | --- | --- | --- | --- |
| **Variável** | **PHQ.1** | | **PHQ.2** | | **PHQ.3** | | **PHQ.4** | | **PHQ.5** | | **PHQ.6** | | **PHQ.7** | | **PHQ.8** | | **PHQ.9** |
| PHQ.1 | 0.000 |  | 1.519 |  | 0.000 |  | 0.000 |  | 0.605 |  | 1.136 |  | 1.092 |  | 0.000 |  | 0.000 |
| PHQ.2 | 1.519 |  | 0.000 |  | 0.000 |  | 1.802 |  | 0.755 |  | 0.880 |  | 0.000 |  | 0.583 |  | 0.000 |
| PHQ.3 | 0.000 |  | 0.000 |  | 0.000 |  | 0.935 |  | 0.699 |  | 0.667 |  | 0.735 |  | 0.675 |  | 1.092 |
| PHQ.4 | 0.000 |  | 1.802 |  | 0.935 |  | 0.000 |  | 0.000 |  | 0.686 |  | 0.000 |  | 0.000 |  | 0.000 |
| PHQ.5 | 0.605 |  | 0.755 |  | 0.699 |  | 0.000 |  | 0.000 |  | 0.265 |  | 0.558 |  | 0.000 |  | 0.000 |
| PHQ.6 | 1.136 |  | 0.880 |  | 0.667 |  | 0.686 |  | 0.265 |  | 0.000 |  | 0.000 |  | 0.849 |  | 1.194 |
| PHQ.7 | 1.092 |  | 0.000 |  | 0.735 |  | 0.000 |  | 0.558 |  | 0.000 |  | 0.000 |  | 1.093 |  | 0.000 |
| PHQ.8 | 0.000 |  | 0.583 |  | 0.675 |  | 0.000 |  | 0.000 |  | 0.849 |  | 1.093 |  | 0.000 |  | 0.295 |
| PHQ.9 | 0.000 |  | 0.000 |  | 1.092 |  | 0.000 |  | 0.000 |  | 1.194 |  | 0.000 |  | 0.295 |  | 0.000 |


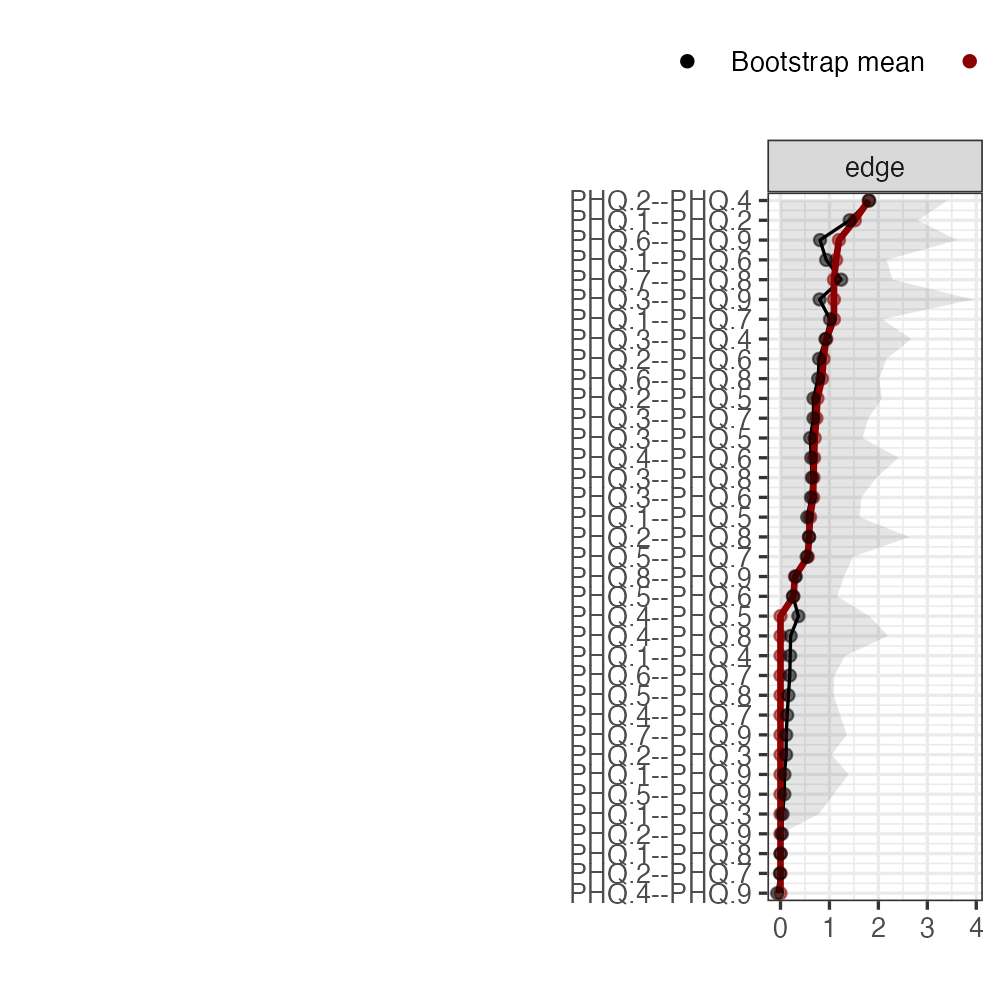

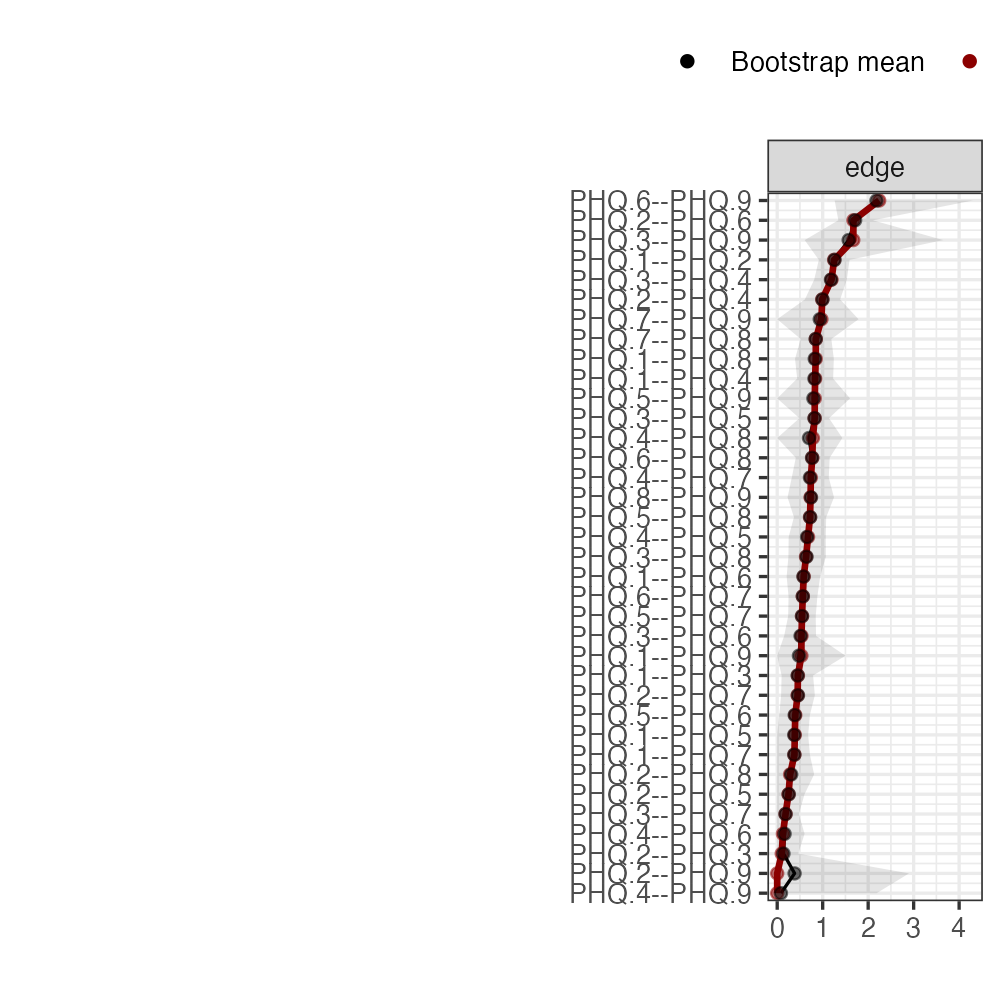


**Figure 1 -** Bootstrap confidence intervals of Edge weights.

Note: Black dots indicate the values of each edge weight, and the gray area represents the 95% confidence interval estimated with a non-parametric bootstrap procedure.

Note: PHQ.1 (anhedonia); PHQ.2 (depressed mood); PHQ.3 (sleep); PHQ.4 (energy); PHQ.5 (appetite); PHQ.6 (guilt); PHQ.7 (concentration); PHQ.8 (motor); PHQ.9 (suicide).


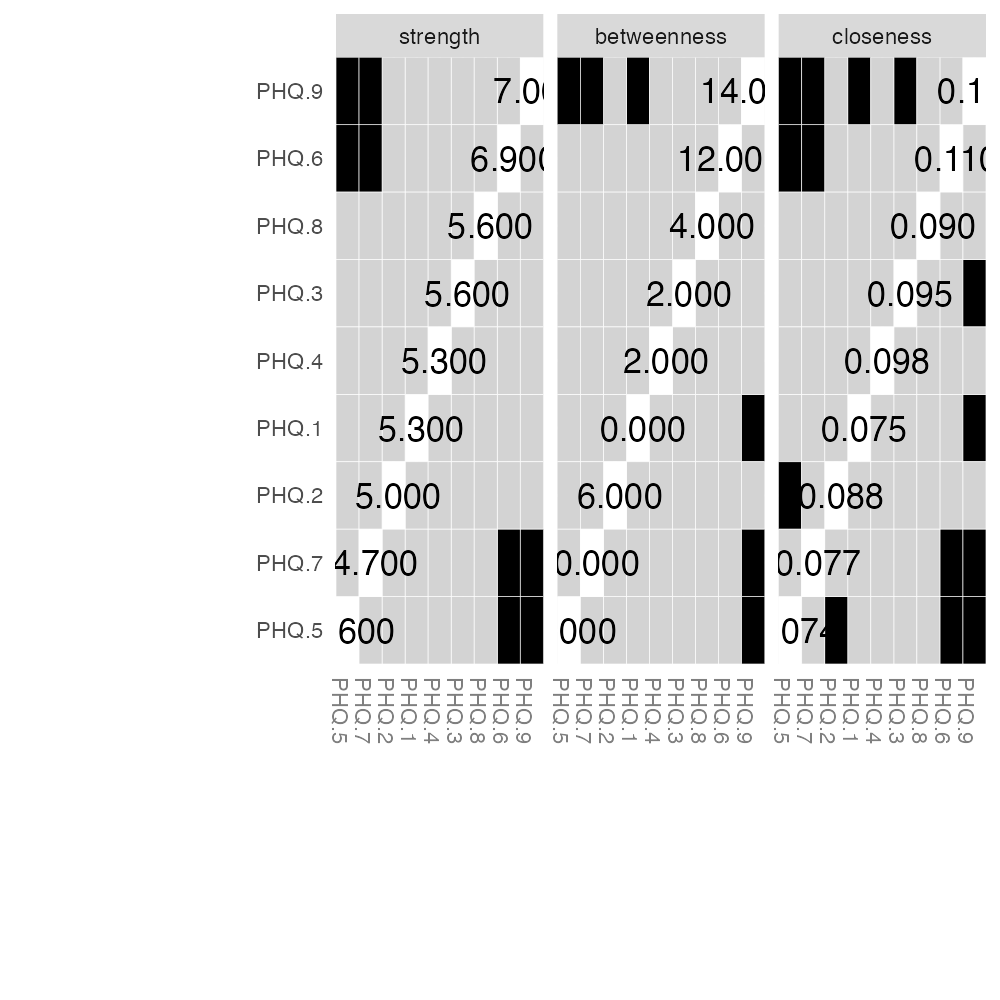

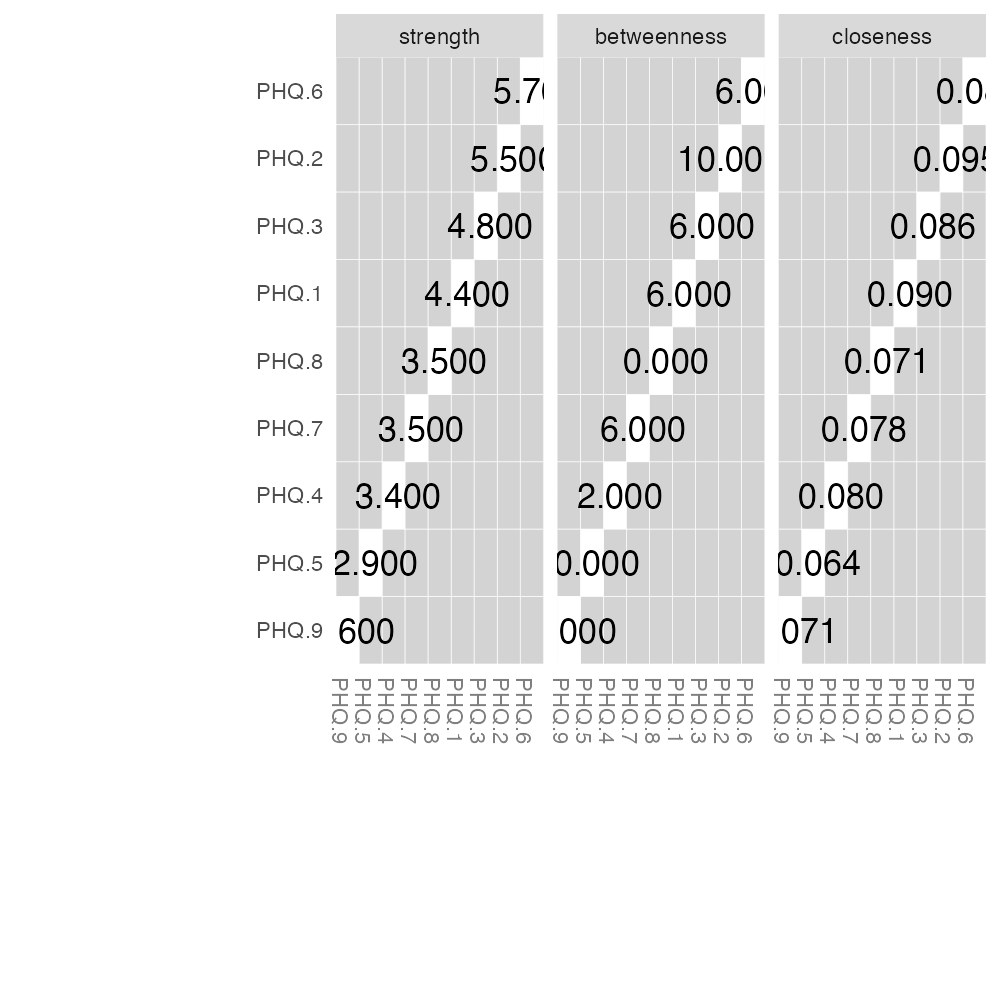


**Figure 2 -** Node strength difference estimation by bootstrap difference test.

**Table 3 –** Standardized centrality index of node centrality degree (strength) for PHQ-9 depressive symptoms in the heterosexual student group and the sexual minority group.

|  | **Heterosexual** | **Sexual minority** |
| --- | --- | --- |
| **Variável** | **Strenght** | **Strenght** |
| PHQ.1 (anhedonia) | -0,336 | 0,291 |
| PHQ.2 (depressed mood) | -0,651 | 1,349 |
| PHQ.3 (sleep) | 0,047 | 0,693 |
| PHQ.4 (energy) | -0,248 | -0,537 |
| PHQ.5 (appetite); | -1,083 | -1,020 |
| PHQ.6 (guilt) | 1,558 | 1,472 |
| PHQ.7 (concentration) | -1,023 | -0,489 |
| PHQ.8 (motor) | 0,079 | -0,472 |
| PHQ.9 (suicide) | 1,657 | -1,287 |
